# “I hardly have a problem […] I have my period quite rarely too”: Female football players’ and their coaches’ perceptions of barriers to communication on menstrual cycle

**DOI:** 10.3389/fspor.2023.1127207

**Published:** 2023-03-23

**Authors:** Max Bergström, Malene Rosvold, Stig Arve Sæther

**Affiliations:** Department of Sociology and Political Science, Norwegian University of Science and Technology, NTNU, Trondheim, Norway

**Keywords:** barriers, player development, junior-to-senior transition, avoidance, one-day seminar

## Abstract

**Introduction:**

Symptoms related to the menstrual cycle (MC) affect the performance level and health of female athletes in various ways. Previous research has reported MC symptoms such as pain, mood disturbance, reduced coordination and competition distraction as well as diminished performance levels and an increased injury risk among female elite athletes. Despite this, the coach-athlete communication related to the female hormonal cycle is limited. The aim of the present study was to explore the perceptions of MC communication in a group of junior elite football players and their male coaches in a case study of one youth football team in a specific club in Norway.

**Methods:**

The study used a qualitative approach with semi-structured interviews. In total, 8 female junior elite players (aged 16-20) from a Norwegian football team and 2 of their male coaches participated in the study.

**Results:**

The data analysis revealed two main communication barriers: 1. interpersonal barriers (e.g., false assumptions about the coach/athletes and social discomfort) and 2. knowledge barriers (e.g., unaware/perceptions of insufficient knowledge levels).

**Discussion:**

As the players seemed to be unaware of their insufficient MC knowledge (e.g., failed to see a connection between the MC and their health and performance level), the coaches perceived their knowledge as insufficient and coped mainly by outsourcing MC communication to female staff and apps. Hence, the MC communication was hindered by both the athletes and the coaches (e.g., mutual avoidance). In line with previous research, this study supports that there is a need for developing effective strategies to overcome the interpersonal barriers and knowledge gaps.

## Introduction

The junior-to-senior transition (JST) (1) is a critical stage in the talent development process of many athletes (2, 3). Players’ career progression to a senior elite level in football is affected by both social (e.g., player–coach–teammate interactions) and psychosocial (e.g., wellbeing) factors (4). The JST is often associated with increasing demands in several life areas (e.g., sport and academic), perceived stress, and an increased risk for injuries (5). Previous research suggest that most coaches are men, both in elite sports (6, 7) and even more within team sports compared to individual sports (8). Since the coaches’ gender and their own coaching experience have an impact on their training philosophies and expectations, the coaches’ gender has been found to be influential on the athletes (7, 9). Additionally, compared to male players, female footballers are underrepresented in the literature (10). Since coaches are known to be the most central socializing agents for young athletes, their views and thoughts are assumably very important for the athletes. This gender hierarchy in sport may affect coaches to view masculine features as the norm, which can influence their coaching methods and understanding for female athletes negatively (7). Hence, female-specific needs during the JST (e.g., the menstrual cycle, MC) might be overlooked (6, 11), seen as a problem, or weakness (7). Furthermore, this may not only inhibit female athletes in their sportily development but also risk their health and wellbeing (11, 12).

Previous research has shown that symptoms related to the MC affect the performance level and wellbeing of many female athletes in various ways (6, 13–16). For example, MC disturbance is one of the most well-known signs of Relative Energy Deficiency in Sport (RED-S), which can impair athlete performance negatively (e.g., increased risk of injury, decreased level of strength and endurance, and reduced training response) (17). Other studies have reported symptoms such as pain, mood disturbance, reduced coordination, and competition distraction (e.g., worry) among senior elite athletes from various sports (16, 18–20). Additionally, Read et al. (15) reported symptoms such as decreased appetite, reduction in sleep quality, recovery and perceived self-confidence, as well as diminished performance levels among elite footballers. Previous research has also shown an association between hormonal fluctuations and anterior cruciate ligament (ACL) injury (21, 22). For example, Martin et al. (23) reported a significant increase (88%) in muscle and tendon injuries (e.g., rupture, tear, strain and cramps) in the late follicular phase compared to other phases of the MC among international footballers. McNamara et al. (19) showed that as much as 65% of 195 female Australian individual sports athletes preparing for the Olympic and Paralympic Games 2020 perceived that their MC affected their performance. To manage their MC, many athletes use hormonal contraceptives (HC) (11, 14, 18, 24). For example, Ekenros et al. (13) reported that 63% from a group of 1,086 athletes used various HC, from which 40% perceived a variety of side effects. Although HCs may affect athlete performance in multiple ways (e.g., weight gain, tiredness, depression, and a decrease of maximal aerobic capacity) (18, 25, 26), the HC knowledge among elite athletes is reported to be low (23). Not surprisingly, only a minority of female athletes considered MC or HC issues when planning their training and competitions (13).

Several recent studies have reported that the athlete–coach communication related to the female hormonal cycle is limited [e.g., (6, 8, 16, 18)]. To improve the current situation, previous studies have urged for educational interventions including, for example, basic terminology and organized discussion forums (6, 11, 18, 27). Such measures are believed to help athletes and their training staff to relate the female hormonal cycle to sport performance and thereby putting it in the same light as other physical functions (18). This can hopefully lower existing communication barriers by increasing awareness and openness (6, 11, 16) as well as correct existing misinformation and misconceptions (12, 18, 20). Höök et al. (11) described this as interpersonal barriers in their study of elite female cross-country athletes and their coaches.

The limited athlete–coach communication related to MC could be explained by the social discomfort of talking about the topic, again related to the gender of the coach (often men) and a lack of specific MC knowledge (11, 27, 28). For example, von Rosen et al. (8) reported that female athletes perceive the knowledge acquired by their male coaches as poor or very poor compared to female coaches. Solli et al. (6) showed that 92% of 140 female athletes (in individual sports) felt that they had insufficient knowledge related to how the hormonal cycle affects athletic performance. Although many of these athletes experienced several MC-related symptoms, only 27% communicated with their coach about their MC (6). Similar findings have been reported in other studies [e.g., (12, 14)]. Additionally, Verhoef et al. (12) suggested five main reasons among female athletes who avoid reporting MC abnormalities (e.g., amenorrhea) to their coaches and training staff: (1) normalization of amenorrhea in elite sport, (2) not expecting the absence of an MC as a problem, (3) shame and taboo, (4) prioritization of sports performance, and (5) denial of the problem. Similarly, Höök et al. (11) found that some elite athletes failed to recognize MC disturbance as a potential health risk, as others believed that there was no need to discuss their MC with their coaches, since they used HC. It was worth noticing that previous research has reported limited MC knowledge levels among coaches as well [e.g., (11, 27)]. Höök et al. (11) described these as knowledge barriers in a study of elite female cross-country athletes and their coaches. Consequently, the combination of social discomfort and insufficient knowledge among female athletes and their coaches may hinder effective MC communication and the incorporation of the MC into the sport-specific practice in both individual and team sports (8).

Given that many previous studies have urged for educational efforts, there is a lack of research that have explored the results of such interventions. Furthermore, previous studies have mainly focused on senior athletes [e.g., (15, 18, 19)] and athletes in individual sports [e.g., (6, 11, 20)]. Since the JST is a critical stage in the talent development process (2, 3) and that the MC may affect the performance level and wellbeing (6, 13–16), it is crucial to explore the experiences of MC communication among junior athletes and their coaches in team sports as well. Knowledge about junior athletes is important since we might expect that they would consider talking about MC more difficult compared to senior athletes. Furthermore, the end of the teens might be a critical period since symptoms related to MC might be more severe for younger athletes compared to senior athletes. Therefore, the aim of the present study was to explore the perceptions of MC communication in a group of junior elite football players and their male coaches in a case study of one youth football team in a specific club in Norway. The team had 6 months earlier been a part of a one-day seminar on MC organized by the club. The importance of the seminar will be discussed in our discussion.

## Methodology

### Participants and data collection

To gain an in-depth understanding of the perceptions and experiences of junior elite female athletes and their coaches’ barriers to MC communication, a qualitative approach was conducted. In the present study, the *Conceptual map of the social support services* model of Bianco and Eklund (29) was applied for the understanding of communication. The model highlights the distinctions between social support activities (e.g., measures/actions done) and social support messages (e.g., the meaning of such measures/actions). Furthermore, the model illustrates the complex processes of how intended support (e.g., from the coach) is perceived by the receiver (e.g., the athlete) depending on the individual’s expectations and perceived needs of social support (e.g., instrumental or relational). Hence, the social support actions and messages may not always match the expectations and perceived needs of the receiver or be interpreted differently from what the sender intended (29), which may affect further actions and communication on specific topics (e.g., the MC). Therefore, exploring how athletes and coaches perceive MC communication as well as their actions taken may help understand potential barriers (or the opposite).

Potential participants were contacted through the team’s assistant coach (coaches) and team captain (players). In total, eight female junior elite footballers (age 18 ± 2 years) and two of their male coaches (assistant coach and physical coach) from the team agreed to participate. The informants were playing or coaching on the highest junior level in Norway. To ensure that the informants would refer to the same case, they were recruited from one football team. The number of participants was considered sufficient in relation to the study aim, sample specificity, and quality of the dialog. This is in line with the concept of “Information power” by Malterud et al. (30). For example, Malterud et al. (30) stated that, “*Information power indicates that the more information the sample holds, relevant for the actual study, the lower amount of participants is needed*” (p. 1753). Our study is positioned within a social interactionist ontology and utilizes an interpretivist approach (31). The focus of this study is on the everyday interactions that occur between individuals, and how the meanings associated with these interactions are managed and transformed through peoples’ interpretative processes as they try to make sense of, and adjust to, their social worlds. The female researcher on the project conducted the interviews, which was intentional since the topic has been considered a taboo topic, especially female athletes talking to male coaches (11). With our social interactionist ontology approach, we considered it lightly that the interviewer and the interviewed would empathize with and identify each other in some way [see (32)].

The data were collected through two semi-structured focus group interviews (players and coaches separately) and three individual interviews (players). The variations in the interview technique were determined by the players’ and coaches’ schedules and availability to participate in the study. Before conducting the interviews, all participants were appraised with the study aim and given all necessary information before obtaining their written consent. All participants were informed that their contribution was voluntary and that they could withdraw from the study at any time during the research process until the article was published. To ensure confidentiality, the participants were given pseudonyms. The interviews took place in the team's sport arena in February and March 2022 and lasted from 25 to 60 min and were held by the second author. The interview guide was inspired by Höök et al. (11) and was organized around the following themes: (1) menstruation and sport, (2) communication, (3) contraceptives and sport, (4) knowledge, and (5) coach–athlete relationship. The interviewer functioned as a facilitator to encourage everyone to contribute, as well as keeping the discussions relevant to the study aim. Since the female hormonal cycle is perceived as a tabooed topic by many athletes and coaches (8, 11, 12), the interviewer used probes in line with Patton (33) to encourage the participants to share their own thoughts and experiences. Examples of such probes were (a) the interviewer started the discussion by sharing her own MC experiences and (b) pointing to recent media coverage of the MC in elite sports. At the end of each probe, the interviewer added relevant questions for each theme such as: (1) “Can you describe how the MC affects you in your athletic career?” (menstruation and sport); (2) “How do you feel about discussing the MC with your coach/athletes?” “Can you recall a specific situation?” (communication); (3) “Do you have any experience with HC?” “How did/does it affect you as an athlete?” (contraceptives and sport); (4) “What additional knowledge would you need about the MC?” (knowledge); and (5) “Tell me about your relationship with your coach/athletes in general” (coach–athlete relationship).

### Data analysis

The aim of the analysis was to identify the different barriers athletes and coaches had encountered in their MC communication. To analyze the interview data, the present study used the six steps of thematic analysis: (1) familiarizing yourself with the data, (2) generating initial codes, (3) searching for themes, (4) reviewing themes, (5) defining and naming themes, and (6) producing the report (34). The first and second authors worked closely with categorizing the raw data. After transcribing the interviews, the first and second authors read the text to get a general sense of the material (step 1). Next, interesting features were bunched into main themes (step 2) (e.g., knowledge) and subthemes (step 3) (e.g., assumptions), based on a deductive analysis based on interpersonal and knowledge barriers. In step 4, all authors reviewed and discussed the themes and subthemes from different research angles and implications. No specific framework for classifying communication barriers were used for the analysis. However, when the authors explored the perceptions of MC communication among the informants, several barriers were discovered from the dataset, also in line with the study of barriers by Höök et al. (11). In the next step, the themes were then refined and labeled into two main themes [(1) interpersonal barriers and (2) knowledge barriers] (step 5). In the final stage of the analysis (step 6), several quotes were chosen to reflect these themes in relation to the study aim and previous research. Additionally, to ensure peer validity, the authors discussed various perspectives and interpretations of the themes throughout data analysis. This is in line with previous literature recommendations (33).

### Ethical statement

The study was conducted in line with the Declaration of Helsinki and approved by Norwegian Social Sciences Data Services (reference nr. 613821).

## Results

**Figure 1:**
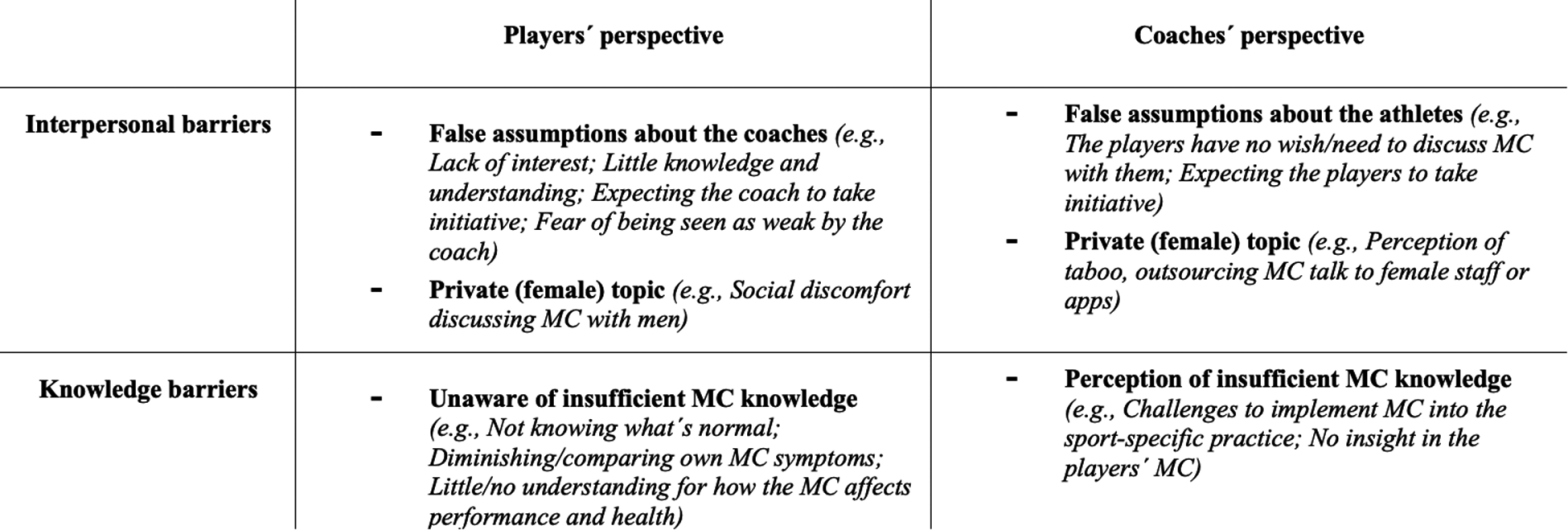
Description of the barriers experienced by the players and coaches based on the thematic analysis.

The qualitative analysis revealed two main barriers faced between female junior footballers and their coaches. The first barrier, *interpersonal barriers*, reflects the players’ and coaches’ perceived challenges for discussing the MC with each other and how this led to the avoidance of MC communication (see Figure 1). The second barrier, *knowledge barriers*, reflects how limited knowledge limits both players and coaches to understand how the MC can affect the players’ sport performance and their health.

### Theme 1: interpersonal barriers

Six months prior to the interviews, both players and coaches had attended a 1-day seminar organized by the club. The seminar was focused on training measures, nutrition, and injury prevention in relation to the MC. The aim of the seminar was to increase the MC knowledge levels and the initiative had been appreciated by both players and coaches. Despite this, they still struggled to communicate with each other about the MC at the time of the interviews. It was worth noticing that both the players and the coaches described to have a close coach–athlete relationship. Yet, discussing the MC was perceived as something private compared to other performance-related factors. Interestingly, the interpersonal barriers were in many cases based on false assumptions from both the players and the coaches. For example, the players assumed that their male coaches were not interested in knowing anything about their MC and that they would not have sufficient knowledge or understanding. Other players believed that the coaches would see them as weak if they would approach them with their MC symptoms:

You don’t feel that they [men] care about it, somehow. One thinks that they are not interested, and then we leave it at that (**A5**).

If you have so much pain and hurt or a lot of discomfort, I think it's difficult to tell a coach because I think he thinks it's strange. You feel that he is not going to respect you **(A7)**.

Consequently, this made the players await the coaches to take the initiative, even though some may have felt a need to discuss their MC. The coaches, however, interpreted the silence from the players as if they had no wish or need to share information about their MC with them, even if they had at least a basic understanding about the MC, as expressed by **C1**:

I have a twin sister, I have a wife, I know. We cannot force them [*the players*]. We cannot make them come up to us and let us know when they have their time of the month. They have to feel comfortable to be able to do it without, I think, us saying it.

Hence, this created a situation in which both players and coaches expected the other to take the initiative and where silence was interpreted as if there was no need to talk to each other about the MC. In addition to the players’ and coaches’ assumptions about each other, the MC was perceived as a private topic. The MC was associated with taboo and social discomfort. For example, the players perceived the MC as a difficult subject to discuss with men in general. Hence, the interpersonal barriers might not have been unique between them and their present coaches. For example, the two following quotes show that the players had experienced MC-related problems (e.g., having to change sanitary napkin or vomiting) but chose to conceal them from their coaches with what they perceived as less discomforting excuses:

At least I've had mostly boys’ and men's coaches who haven’t said anything about this at all. And then it becomes a bit unpleasant to say “I have to go to the bathroom,” or I don't really have to go to the bathroom, I just have to change tampons. You don’t like to say that to your coach if he's a man (**A3**).

When we played on another team earlier, we also had a male coach. That's when I threw up. The next practice when I came and he asked how it went and that I had thrown up, I just said that I must have eaten something bad because I didn’t want to say that the reason was my period (**A8**).

The coaches coped by outsourcing MC talks to female staff or MC apps. Their coping strategies may indicate that discussion of MC directly with the players was associated with social discomfort but also it reflects a need to help their athletes:

I feel that it is easier for the players to talk to her [*the female coach*]. We also have a female fitness coach, and that is important … so we try to manage that [*having female staff*], and prioritize that [the MC] (**C1**).

[…] if they want to report it to us [*about their MC*], they can *via* that app. That goes to our physical coach, who is female. We could possibly log the cycle of every player. That is a lot of work, but if they want they can do it (**C2**).

Interestingly, both the players and the coaches seemed to be aware that the MC was relevant to the sport practice at least to some extent but still struggled to find ways to initiate conversations about it. Hence, this left them waiting for each other. For example, as mentioned in the second quote from **C2**, he says: “…*if they want, they can do it*,” thereby leaving the initiative to the players. However, since the players seemed to conceal MC-related symptoms from their coaches, as mentioned by **A3** and **A8**, this also hindered MC communication by leaving the coaches unknowing.

### Theme 2: knowledge barriers

The second barrier that inhibited MC communication was limited MC knowledge levels among both the players and the coaches. For example, some players failed to recognize MC disturbance as a symptom relevant to their performance and health, as expressed by **A4**:

I hardly have a problem […] I have my periods quite rarely too … So I am bothered very little compared to many others, but I, who am bothered so little, have had no need to talk about it. I get through a week where I’m a bit tired without needing to put anything up after that.

Additionally, **A4** compares her own symptoms to what she thinks her teammates might experience and concludes that they probably are worse off than her. Because of her perception of just having minor or no MC problems, she sees no need to share the information with the coaches. Other athletes seemed to believe that the MC did not concern them anymore, since using HC. Instead, she diminishes the perceived MC symptoms and continues with the training as normal. This is similar to **A6,** who describes her experiences with MC symptoms:

I am very lucky that I have not suffered from pain. I can feel it occasionally in my stomach and lower back, but it has not been a problem […] You are afraid of bleeding, especially during training and the like. Now I don’t think about it too much. It's only if my back hurts that I get a little stiffer after training. Right after […] we fortunately have black shorts […] We have white away shorts, and then I think more about it.

**A6** starts with expressing a contradiction. In the first sentence she says that she is lucky because she does not have any MC-related pain, but still admits it in the next sentence. Just like **A4, A6** seemed to believe that her problems were not big enough or irrelevant to share with the coach staff. Additionally, she mentions implicitly that the fear of bleeding through her white shorts affects her concentration during trainings and games. Although she was aware of the distraction this meant to her during practice and games, she failed to make the connection that this might affect her performance level. Hence, she did not approach the coaches with the information.

The coaches felt that they would not know how to implement the perceived MC symptoms in a team setting and their sport-specific practice, for example, as **C2** sees challenges in logging the MC or individualizing the training for each of the 27 players. The perception of having insufficient MC knowledge was shared by **C1**:

I think one of the difficulties in football is that we are a team of 27 players, all individuals […] In some way we try to log their monthly cycle, but it is hard in a team setting to put menstruation cycle in to account in training (C2).

For me as a man, the barrier must be broken down for us to get the fullest knowledge and understanding and appreciation of how to work with it [*the MC*] in the best way we can […] My knowledge is not enough, because we need to be having more conversations (C1).

Interestingly, **C1** is aware that there is a barrier between him and the players. Furthermore, he sees having MC conversations as a key to gain more knowledge and to improve his understanding in order “*to work with it in the best way*.” Yet, as mentioned in theme 1, both the coaches and players avoided initiating such talks. As the players seemed to be unaware of their insufficient MC knowledge (e.g., failed to see a connection between the MC and their health and performance level), the coaches perceived their knowledge as insufficient. For example, they felt unsure how to implement the MC into the sport-specific practice. Almost contradictory, they believed, on the one hand, that discussing the MC with their players would enhance their knowledge, as expressed by **C1**. On the other hand, they seemed to avoid it because of the perceived lack of MC knowledge. Therefore, avoiding MC communication also kept them unaware of what the actual MC issues were such as fear of bleeding through their shorts. Consequently, in combination with the perceived interpersonal barriers, the *status quo* (e.g., no MC communication) was maintained by both the players and the coaches.

## Discussion

The aim of the present study was to explore the perceptions of MC communication in a group of junior elite football players and their male coaches in case study of one youth football team in a specific club in Norway. The data analysis revealed two main communication barriers: (1) interpersonal barriers (e.g., false assumptions about the coach/athletes and social discomfort) and (2) knowledge barriers (e.g., unaware/perceptions of insufficient knowledge levels). Similar to Höök et al. (11), our study focused on both the athletes’ and coaches’ perspectives. In line with previous research showing interpersonal barriers [e.g., (6, 8, 16, 18)], the present study showed that the coach–athlete MC communication was limited even though the athletes reported several MC symptoms.

Previous studies have suggested educational interventions including, for example, basic terminology and organized discussion forums as an important step to enhance MC communication (6, 11, 18, 27). Interestingly, both the athletes and their coaches in the present study had attended at a 1-day MC seminar organized by the club. Even so, there was no coach–athlete MC communication or perceived changes in how the club worked with MC issues 6 months later, without discussing the quality or the length of the seminar. The data analysis did regardless reveal that the interpersonal barriers were partly a consequence of false assumptions and prejudices. As the athletes assumed that the coaches would not want to be bothered with MC-related problems or that they would not be able to understand them, the coaches interpreted their silence as if they had no will or need to discuss MC issues. The findings show that the coaches had more understanding and willingness to help than the athletes expected. In the present study, the coaches coped by outsourcing MC matters to female staff members and MC apps where they had no insight. Although previous research suggest that the MC should be monitored just like the athlete’s training load, recovery, wellbeing, and injuries to promote the long-term development (35), it can be discussed if their coping strategy maintained the interpersonal barriers since it also meant avoiding direct MC communication with the athletes. For example, their lack of direct involvement might have been interpreted as a lack of interest by the athletes. Furthermore, this may also indicate a mismatch between the social support actions and the intended social support messages (28). Temm et al. (35) argued that whichever monitoring method is applied, it is crucial that it can be individualized, is affordable, and easy to implement. Here, possibly combined with other protocols, direct coach–athlete communication can be an effective and inexpensive way to prevent ACL injury and RED-S among female athletes.

A reason for the interpersonal barriers found in this study might be that elite sports is embedded in the normalized “culture of risk” (36), found both among female and male football players (37). The expectation of always striving for success and accepting health risks are internalized by both athletes and coaches (37). Within the literature, the risk of injury studies show that players are unwilling to “play hurt”—risk being stigmatized, isolated, and ignored by coaches (38), and consequently have been found to play a pivotal role in decisions whether to compete while injured (39). This could be related to the present study since some athletes feared of being seen as weak by the coaches if they would approach them with their MC symptoms. Even though our study did not focus on the risk of injury, this may indicate that they were concerned about how their development opportunities (e.g., risking getting dropped out of games) would be affected. However, ignoring or concealing MC symptoms (e.g., MC disturbance, pain or sickness) may be a counterproductive strategy in the long-term performance development perspective (e.g., reduced performance level, increased injury risk, or developing RED-S) (15, 17).

Previous research has shown that many athletes perceive that their MC and the knowledge about it affect their performance (19). Yet, only a minority discuss this with their coach (6, 12, 14). Similar to other studies [e.g., (11, 27, 28], the lack of MC knowledge and communication among the athletes and coaches was also affected by social discomfort (e.g., shame and taboo) and the gender of the coach. For example, the athletes believed that men in general (including their coaches) were not interested in the MC. This is in line with von Rosen et al. (8), who reported that female athletes perceive the knowledge acquired of their male coaches as poor or very poor compared to female coaches, indicating a knowledge barrier. However, it can also be speculated if the players perceived a gender hierarchy (7) in their sport that might have affected them to view masculine features as the norm, and thereby the MC as an abnormality or weakness. Hence, this may be another explanation for why some of the athletes feared that the coaches would see them as weak if they would approach them with MC issues.

The data analysis also revealed that lack of MC-specific knowledge limited the coach–athlete communication as well, indicating that the interpersonal barriers and knowledge barriers were impacting on each other. Although the athletes reported symptoms such as MC disturbance, pain, and sickness, they did not report this to their coaches. Two possible explanations for this phenomenon are that they (a) failed to recognize the symptoms as something abnormal and relevant to their performance and health and (b) compared their symptoms with what they believed others experienced. This is in line with Verhoef et al. (12) who reported athletes’ prioritization of sport performance, normalization of amenorrhea, and denial of it as a problem that hinders MC communication, based on lack of knowledge. Additionally, some athletes using HC seemed to believe that the MC did not concern them anymore. Hence, in line with Bianco and Eklund (28), this may have affected the perceived need of MC-specific social support from their coaches. Similar findings have been seen in other studies [e.g., (11)]. Notably, the players had attended a 1-day seminar that focused on training measures, nutrition, and injury prevention in relation to the MC together with their coaches. Such educational interventions are encouraged in the literature since they have the potential to lower existing communication barriers by increasing awareness and openness (6, 11, 16) as well as correct existing misinformation and misconceptions (12, 18, 20).

Even though the players and the coaches described the seminar as a positive initiative by their football club, a limitation of this study is that exact content and working methods used in the seminar were unknown to the researchers. However, based on the findings as already stated, clearly a 1-day seminar does not seem to be enough, indicating the need for more educational interventions, even though it also depends on the content of such interventions. For example, the present study showed a need for educating athletes about how MC symptoms can affect sport performance and the potential health risks of ignoring them. Yet, the main challenge to overcome seemed to be the interpersonal barriers. In contrast to the players, who seemed to be unaware of their insufficient MC knowledge, the coaches perceived their knowledge as insufficient. In the present study, we found a paradox that exemplifies the challenges perceived by the coaches. On the one hand, the coaches believed that discussing the MC with their athletes would enhance their knowledge. On the other hand, they seemed to avoid it because of the perceived lack of MC knowledge or players sharing their experiences. Furthermore, they felt unsure how to implement the MC into the team sport setting (e.g., social support actions). Here, one critical question is how they would implement the MC into their sport practice if they did not find out what the perceived MC issues among the players were. Rather, it seemed that avoiding or outsourcing MC communication kept them unaware of MC issues, such as fear of bleeding when playing with white shorts, which would have been a relatively easy thing to “fix” (e.g., changing the color of the team shorts). Furthermore, if athletes and coaches would be able to communicate the MC, it may also help them to identify where the knowledge gaps are and plan future educational interventions together based on this. Yet, how could coaches and athletes be aware of the importance of discussing the MC without at least a basic understanding of the MC? Therefore, future studies could explore the best place to start. In line with previous research (6, 11, 12, 16, 18, 20), this study supports that there is a need for developing effective strategies to overcome the interpersonal barriers and knowledge gaps. Since our study shows that these barriers were maintained by both the players and coaches, an active engagement from both athletes and coaches, as well as support from their sport clubs and sport federations may be necessary in changing the “status quo.” Even so, since coaches are known to be the most central socializing agents for young athletes and their views and thoughts are assumably very important for the athletes, one might expect the coaches to be the initiative taker on this issue. On a deeper level, this may also mean a continued work with gender hierarchies (7) and gender biases (9) in sport (e.g., in research). This will hopefully contribute to enhance sport performance and injury prevention, as well as female athletes’ health and wellbeing.

### Limitations

There are some limitations in the current study that must be considered. The use of both focus group and individual interviews might be considered a limitation since the focus group participants might have spoken more freely if they were interviewed individually. On the other hand, it might be that they spoke more freely when they experienced that the other players or coaches open on a difficult topic to discuss. The use of a female researcher was intentional because of the topic and the group of youth athletes. Doing qualitative research and adopting a social interactionist ontology and interpretivist epistemology used in our interviews enabled us to frame our interviews as a relational space. This meant that both the participants and the interviewers could explore themes together and co-construct knowledge (31). This might have meant that the interviewed had a strong voice if the interviewer and the interviewed empathized and identified with each other [see (32)], which we would say was the case in the present study. The taboo topic in the paper might be an obvious reason since the female researcher and the participants could talk about a topic, as shown in our results, which is considered difficult to talk about, in our study described as interpersonal barriers to talk to their male coaches. The use of probes in the interviews had the intention to let the interviewed talk about and relate to how they talked about MC was important in their everyday interactions with their coaches. Another limitation could be related to the limited education the coaches and players received through a 1-day seminar, which naturally also must be considered in terms of both the coaches’ and players’ knowledge on the topic. A third limitation was that more detailed information about the athletes’ training and performance level, training hours, menstrual cycle lengths and use of hormonal contraceptives, the educational level of the coaches, as well as demographic data and details about the seminar are missing. This could have added information to the findings and enriched the discussion.

## Conclusion

The main findings of the study indicate that the players seemed to be unaware of their insufficient MC knowledge (e.g., failed to see a connection between the MC and their health and performance level), while the coaches perceived their knowledge as insufficient and coped mainly by outsourcing MC communication to female staff and apps. Overall, it could be argued that the MC communication was hindered by both the players and the coaches (e.g., mutual avoidance). These findings are in line with previous research mostly on individual athletes, supporting that there is a need for developing effective strategies to overcome the interpersonal barriers and knowledge gaps, also within team sports. This will hopefully enhance female athletes’ sport performance and injury prevention, as well as their health and wellbeing.

## Data Availability

The raw data supporting the conclusions of this article will be made available by the authors, without undue reservation.
